# Dynamic modulation of optical potential wells and rotating nanoparticles by radiation force

**DOI:** 10.1016/j.isci.2026.115169

**Published:** 2026-02-27

**Authors:** Jingjing Su, Nan Li, Chuyun Jin, Xingfan Chen, Huizhu Hu

**Affiliations:** 1 School of Transportation Management, Zhejiang Institute of Communications, Hangzhou 311112, China; 2 Key Laboratory of Modern Optical Instrumentation, College of Optical Science and Engineering, Zhejiang University, Hangzhou 310027, China

**Keywords:** Physics, Applied sciences

## Abstract

Flexible adjustment of optical trap stiffness and the photoinduced rotation effect of multiple optical traps have broad application prospects in the quantum field. Research has shown that a flexible and controllable array of Gaussian optical traps offers the advantages of adjustable trap positions, the ability to rotate nanoparticles, the generation of distinct beam shapes through trap stiffness modulation, and simultaneous trapping of two types of nanoparticles with different refractive indices. Based on the distribution formula of the array of Gaussian beams, this study establishes the dynamic regulation of the gradient fields of arbitrary single or multiple beams within a multi-beam optical trap, thereby enabling particle rotation manipulation. This manipulation depends solely on the optical field properties of a single specialized beam.

## Highlights

• Joint flexible control of multiple beams realized and used to rotate nanoparticles
• Use of beam gradient fields in optical traps for particle manipulation
• Simultaneous capture of two types of nanoparticles with different refractive indices
• Targeted beam shapes achieved through trap stiffness modulation

## Graphical abstract

**Figure:**
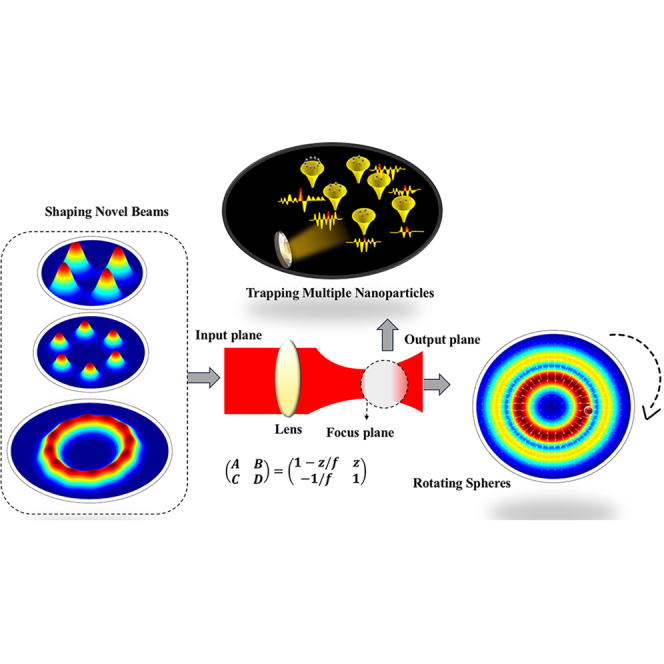

## Introduction

Optical tweezers use the force of light radiation to trap or confine particles, wherein lasers generally output Gaussian beams.^1^^,^^2^^,^^3^ Typically, optical field control can generate structured light fields with a non-uniform amplitude, polarization, and phase distribution, yielding a special capture effect and improving the capture performance of optical tweezers.^4^^,^^5^^,^^6^^,^^7^^,^^8^^,^^9^ Therefore, appropriately structured light fields can be used to perform complex operations on particles.^8^^,^^10^^,^^11^ Currently, new spatial structure light fields, such as non-diffracting, vector vortex, and self-accelerating beams exhibiting non-diffracting, self-bending, self-repairing, and spin-orbit angular momentum coupling characteristics, have been successfully applied in the fields of optical micromanipulation, optical fibers, optical communication, and holographic displays.^12^^,^^13^^,^^14^^,^^15^^,^^16^^,^^17^

Since Sato first realized the light-induced rotation of nanoparticles in a laser-based optical trap by rotating high-order Hermite-Gaussian light, light-induced rotation has attracted extensive attention from researchers worldwide.^10^^,^^18^ Currently, the rotational speed of particles in vacuum optical tweezers reaches 6 GHz, and the torque detection sensitivity reaches $10^{-29}\;N\cdot m/\sqrt{\text{Hz}}$.^19^^,^^20^ Research on photoinduced rotation has great potential in the field of weak force and quantum geometric phase, such as Casimir force and nanometer magnetic poles.^21^^,^^22^ Traditional optical tweezer technology can only produce one optical trap for operation and cannot realize the dynamic modulation of trapping potentials and rotating particles, limiting the operating efficiency of optical capture. Increasing the number of optical traps and adjusting optical trap stiffness are viable options to overcome these limitations, and multi-position optical tweezers have been extensively studied. Specifically, controlling optical trap stiffness provides a method to study the fundamental fluctuation theorems involved in equilibration from nonequilibrium initial states.^23^^,^^24^^,^^25^ Several methods have been reported to capture and operate multiple nanoparticles; these include using nano-piezoelectric transitions to design scanning optical tweezers,^26^ acousto-optic modulators to achieve beam deflection and combination,^27^ and computer programming to achieve multiple time-division multiplexing optical well arrays with complex shapes.^28^

Holographic optical tweezers can dynamically modify any optical point well array in real time and effectively study the particle multi-body interaction in different optical wells.^29^^,^^30^ Currently, the algorithms used for hologram generation include non-iterative algorithms and iterative algorithms. Davis J A proposed a method to modulate the light field by randomly generating masks for creating point optical traps (RM algorithm), This algorithm offers high computational speed but suffers from low optical trap uniformity and diffraction efficiency, with trap quality deteriorating as complexity increases.^31^ Liedener J developed an approach to generate optical fields by superimposing grating and lens phases (S algorithm), yet it tends to produce additional “ghost traps,” which reduce the intensity and uniformity of the target traps.^32^ Lesem L B proposed generating optical traps through the random superposition of phase distributions (SR algorithm), but this method exhibits low modulation efficiency and results in indistinguishable trap arrays.^33^ Non-iterative algorithms are suitable for rapid trap generation but yield low trap quality; thus, researchers have proposed iterative algorithms to create high-quality optical traps. The GS algorithm is a classic iterative phase retrieval technique. Curtis applied the GS algorithm to compute holographic phase patterns and introduced a spherical (lens) phase factor into the target optical field to generate high-precision three-dimensional optical traps.^34^ Although such implementations achieve high accuracy, they suffer from low computational efficiency and a tendency to converge to local optima. Dufresne E R developed an improved iterative algorithm capable of generating complex trap distributions (AA algorithm), though its computational efficiency still requires optimization.^35^ Leonardo R D incorporated weight factors into the GS algorithm to enhance trap uniformity (GSW algorithm), but this introduces a trade-off between computation time and control precision.^15^ Researchers have further modified classical algorithms to improve the reconstruction quality of target light fields and increase computational speed. For light fields with continuous intensity distribution, the computational cost of using either non-iterative or iterative algorithms increases significantly due to the inherent complexity of such fields. Both types of algorithms require extensive computations to simulate and adjust every point in the light field to achieve the desired distribution. As light field complexity grows, the volume of data to be processed and the number of computational steps increase correspondingly, leading to higher computational costs. Iterative algorithms demand repeated iterations until a satisfactory light field distribution is achieved, which substantially elevates the computational burden when handling continuous light fields.

The beam proposed herein enables the joint realization of photoinduced particle rotation and optical trap stiffness regulation in optical tweezers. An Array of Gaussian optical tweezers can achieve independent regulation of the intensity of each optical trap in the array by loading the phase template of an array of Gaussian beams onto a spatial light modulator (SLM), without relying on computational holography. Meanwhile, dynamic rotational control of the overall or local phase patterns can be realized. Additionally, the array of Gaussian beams proposed in this article can be generated using a metasurface. Compared with the relatively large-volume optical system required for holographic optical tweezers, this method offers the advantages of small size, high integration, and easy modulation. Furthermore, we demonstrate that the amplitude of the gradient force and position of the capture point can be obtained by changing the number of nanoparticles in the optical trap and the distance from the trap center using the optical field of the array of Gaussian beams distribution formula. Our results reveal that a flexible and controllable array of Gaussian beams offers the advantages of an adjustable capturing position, distinct beam profiles, and the ability to trap two types of nanoparticles at different refractive indices.

### Field distributions of an array of Gaussian beams

Here, we introduce certain essential mathematical notations. The rotation of angle *θ* on the Cartesian coordinate system is expressed as $R_{\theta }\left(x,y\right)=\left[\begin{array}{cc} \cos\;\theta & -\sin\;\theta \\ \sin\;\theta & \cos\;\theta \end{array}\right]\left[\begin{array}{c} x\\ y \end{array}\right]$, which preserves the length and inner product of vectors. For any function *f*(*x*,*y*), the function after rotation is *f*(*R*_*θ*_(*x*,*y*)) = *f*(*xcosθ*-*ysinθ*,*xsinθ*+*ycosθ*). This is denoted by *R*_*θ*_(*f*), which is the induced action on functions. A function *f*(*x*,*y*) is invariant under rotation if *f* = *R*_*θ*_(*f*). To obtain a function with maxima located at the vertices of a regular polygon, we consider the action of *Z*_*n*_, the cyclic group of order n. Let *θ*_*k*_ = 2*kπ*/*n*(*k* = 0,1,2, …,*n*-1). Under multiplication, these elements form the group *Z*_*n*_. By applying these elements to a function *f*, we obtain $R_{\theta_{k}}\left(f\right)$, *k* = 0,1,2, …,*n*-1. Then, $\sum_{k=0}^{n-1}R_{\theta_{k}}\left(f\right)$ is invariant under the action of *Z*_*n*_.

Consider a fundamental Gaussian beam with linear polarization at the incident plane z = 0, Therefore, $E\left(x_{1},y_{1},0\right)=A_{0}\;\exp\left(-\left({\left(x_{1}-l\right)}^{2}+{\left(y_{1}-l_{1}\right)}^{2}\right)/w_{0}^{2}\right)$, where *A*_0_ is determined by the input power *P*, and *l* and *l*_1_ are the translation factors used to control the beam. Considering the action of *Z*_*n*_ on Gaussian functions, we obtain the electric field as follows:

(Equation 1) $$E\left(x_{1},y_{1},0\right)=A_{0}\sum_{k=0}^{n-1}G_{k}\;\exp\left(-\frac{{\left(x_{1}\;\cos\;\theta_{k}-y_{1}\;\sin\;\theta_{k}-l\right)}^{2}+{\left(x_{1}\;\sin\;\theta_{k}+y_{1}\;\cos\;\theta_{k}-l_{1}\right)}^{2}}{w_{0}^{2}}\right)=A_{0}\sum_{k=0}^{n-1}G_{k}\;\exp\left(-\frac{{\left(x_{1}-\left(l\;\cos\;\theta_{k}+l_{1}\;\sin\;\theta_{k}\right)\right)}^{2}+{\left(y_{1}-\left(-l\;\sin\;\theta_{k}+l_{1}\;\cos\;\theta_{k}\right)\right)}^{2}}{w_{0}^{2}}\right)$$

Therefore, *E*(*R*_*θ*_(*x*,*y*)) = *E*(*x*_1_,*y*_1_)(*k* = 0,1,2, …,*n*-1), implying that *E*(*x*_1_,*y*_1_)is invariant under *Z*_*n*_ when *G*_*k*_ is a constant factor in a Cartesian coordinate system. The maxima of *E*(*x*,*y*) is invariant under the action of *Z*_*n*_; hence, they lie at the vertices of a regular n-polygon. This type of regular n-polygon beam is the object of this study. For simplicity, consider *l*_1_ = 0. Based on the above principles, the electric field distribution of the flexible and controllable array of Gaussian beams at z = 0 is as follows:

(Equation 2) $$E\left(x_{1},y_{1},0\right)=A_{0}\sum_{k=0}^{n-1}G_{k}\;\exp\times \left(-\frac{{\left(x_{1}-l\;\cos\;\theta_{k}\right)}^{2}+{\left(y_{1}+l\;\sin\;\theta_{k}\right)}^{2}}{w_{0}^{2}}\right)$$

where n is the number of generated optical wells, *w*_0_ is the waist width of the Gaussian beam, and *k*_0_ is the wave number related to the wavelength *λ*_0_ of the input light, such that *k*_0_ = 2*π*/*λ*_0_. Among them, *G*_*k*_ is the constant factor of the intensity of any beam in the array of Gaussian beams. In the following simulation, without special explanation, we set *G*_*k*_ as the all-one matrix. For this study, we selected *f* = 5 *mm*,*w*_0_ = 5 *mm*, *P* = 1*W*, and *λ*_0_ = 1064 *nm*.

Using the Huygens-Fresnel diffraction integral with the ABCD optical system, we can describe the generated electric field distribution as follows^36^:

(Equation 3) $$E\left(x,y,z\right)=\frac{i}{\lambda B}\exp\left(ikz\right)\iint dx_{1}dy_{1}E\left(x_{1},y_{1},0\right)\exp\left(-\frac{ik}{2B}\left[A\left(x_{1}^{2}+y_{1}^{2}\right)-2\left(xx_{1}+yy_{1}\right)+D\left(x^{2}+y^{2}\right)\right]\right)=c_{1}\left(\frac{\pi }{a}\right)\sum_{k=0}^{n-1}G_{k}\;\exp\left(\left(b_{1}^{2}+b_{2}^{2}\right)/4a\right)$$

where

(Equation 4) $$c_{1}=\frac{i}{\lambda B}\exp\left(ikz\right)\exp\left(-\frac{ikD}{2B}\left(x^{2}+y^{2}\right)\right)\times \exp\left(-\frac{l^{2}\;\cos\frac{2k\pi }{n}}{w_{0}^{2}}-\frac{l^{2}\;\sin\frac{2k\pi }{n}}{w_{0}^{2}}\right)\text{,}$$

(Equation 5) $$a=\frac{1}{w_{0}^{2}}+\frac{\text{ikA}}{2B},b_{1}=\frac{2l\;\cos\frac{2k\pi }{n}}{w_{0}^{2}}+\frac{\text{ikx}}{B},b_{2}=\frac{2l\;\sin\frac{2k\pi }{n}}{w_{0}^{2}}+\frac{\text{iky}}{B}\text{.}$$

Note that Equations 3, 4, and 5 apply only to the field distribution of an array of Gaussian beams passing through the aperture-free first-order ABCD optical system. For optical systems with an aperture, the influence of the aperture must be included. By translating and rotating the fundamental Gaussian beam in the initial incident plane, the optical field model we proposed can be extended to arbitrary geometric arrays.

**Figure 1:**
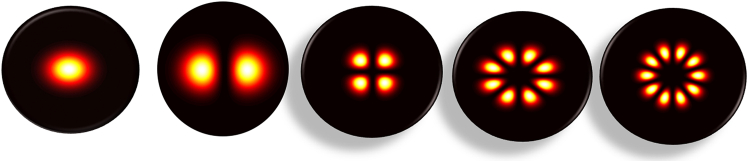
Light intensity distributions of an array of Gaussian beams (*n* = 1, 2, 4, 8, 10) at *l* = *l*_1_ on the front focal plane under the initial electric field

**Figure 2:**
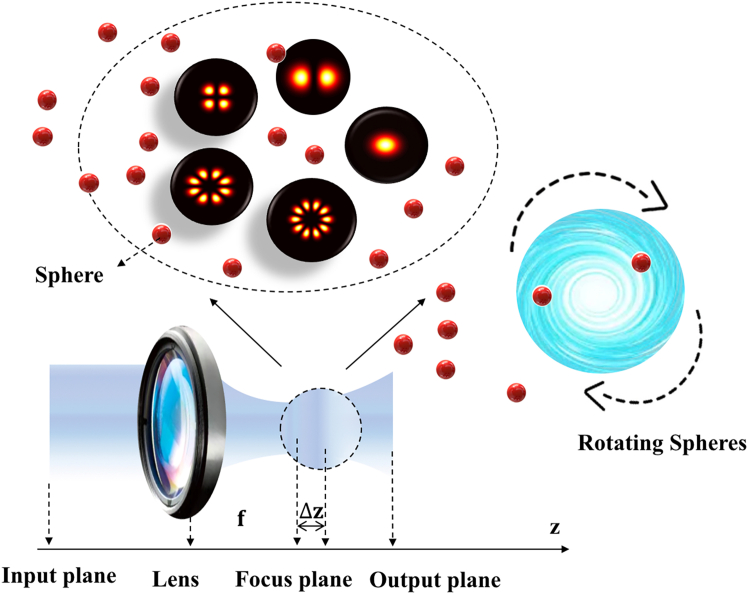
The transmission principle of an array of Gaussian beams

Figure 1 illustrates the distribution of an array of Gaussian beams on the front focal plane before beam incidence. As shown in the figure, the array of Gaussian beams is arranged in a regular polygonal pattern. To fully leverage the advantages of Gaussian array optical traps, a precise design of the optical system is required. By employing focusing lenses and other optical components, the array of Gaussian beams can be concentrated into a miniature array of optical traps. Within these traps, the attractive force of the beams can be utilized to capture and manipulate nanoparticles. Figure 2 simulates the trapping behavior of nanoparticles in an array of Gaussian optical traps, and provides a schematic diagram showing the propagation of an array of Gaussian beams along the z-direction through a focusing objective optical assembly.

### Optical properties of a flexible and controllable array of Gaussian beams

Here, we define the transverse and axial planes of the light field. The plane parallel to the focal plane of the captured nanoparticles (i.e., the x–y plane) is the transverse plane, and the axial plane (i.e., the x–z or y–z plane) is perpendicular to the focal plane.

We consider that the array of Gaussian beams is focused by a high numerical aperture lens in the optical well. In the paraxial approximation framework, the input and output planes to the transmission matrix can be expressed as *A* = 1-*z*/*f*,*B* = *z*,*C* = −1/*f*,*D* = 1, where *A*, *B*, *C*, and *D* are the transfer matrix elements of the optical system, *z* is the axial distance from the lens to the output plane, and *f* is the focal length of the focusing objective. The array of Gaussian beams focuses at the focal point of the lens, and a single focal point is present on the focusing plane. Following emission from the focal plane, the transmission state of multiple beams is restored.

### Radiation force generated by an array of Gaussian beams

When the radius of the nanoparticles is significantly smaller than the wavelength of light, and the nanoparticles in the optical trap are excited by the electromagnetic field to form electric dipoles, the radiation force of the array of Gaussian beams exerted on the nanoparticles can be calculated using the Rayleigh scattering model.

The polarizability *α* is defined as follows:

(Equation 6) $$\alpha =4\pi r^{3}\frac{\varepsilon -\varepsilon_{m}}{\varepsilon +2\varepsilon_{m}}$$

where *r* is the radius of the particle, and *ε* and *ε*_*m*_ are the dielectric constants of the particle and medium surrounding the particle, respectively. The optical force acting on Rayleigh particles can be rigorously decomposed into three components, i.e., the gradient force, the scattering (radiation pressure) force, and the spin curl force (non-conservative force). Based on the generation mechanism of the spin curl force (non-conservative force), for the array of Gaussian beams—a typical uniformly polarized light field—the contribution of the spin curl force to the optical force acting on Rayleigh particles is strictly zero. Under these circumstances, the total optical force can be simplified to^37^^,^^38^:

(Equation 7) $${\vec{F}}_{grad}=\frac{1}{4}\varepsilon_{0}\varepsilon_{m}\text{Re}\left(\alpha \right)\nabla \left|\vec{E^{2}}\right|$$

(Equation 8) $$\vec{F_{scat}}=\frac{\varepsilon_{0}{\varepsilon_{m}}^{3}k_{0}^{4}}{12\pi }\left|\alpha^{2}\right|\left|\vec{E^{2}}\right|\vec{z}$$

where *ε*_0_ and *k*_0_ are the dielectric constant and wave number in vacuum, respectively. In subsequent calculations, we consider a particle of radius *r* = 20 nm. $\varepsilon_{m}=n_{m}^{2}$ and $\varepsilon =n_{p}^{2}$ denote the dielectric functions of the Rayleigh particle and the surrounding medium, respectively. The ambient refractive index is *n*_*m*_ = 1.33 (i.e., water), and a high and low-refractive-index particle exhibits *n*_*p*_ = 1.592 (i.e., polystyrene) and *n*_*p*_ = 1 (i.e., air bubble), respectively.

**Figure 3:**
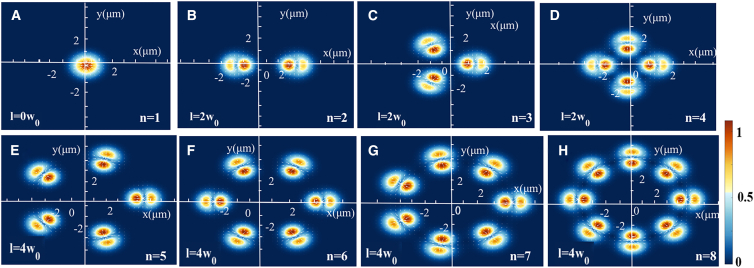
Gradient force acting on the nanoparticles in the xy-plane with different *n* values and translation distance *l* in the transverse plane of the optical trap with z = 5.5 μm (A–H) Show the distribution of the output beams of the generated n = 1–8 array of Gaussian beams on the focal plane through the focusing system. (A) illustrates a single Gaussian beam generated at translation distance *l* = 0*w*_0_, (B–D) illustrate array of Gaussian beams generated at translation distance *l* = 2*w*_0_, and (E–H) illustrate array of Gaussian beams generated at translation distance *l* = 4*w*_0_. Note that (C–H) indicates that the output beams of the array of Gaussian fields are arranged on the output plane in the form of a regular polygon. The color bar with a normalized range of 0–1 characterizes the magnitude of the optical field gradient force. The color variation directly reflects the spatial change rate of optical intensity, with different colors indicating the specific magnitude of the optical intensity gradient.

By considering different values for the parameter n of the beam electric field and translation distance *l*, different numbers of optical wells can be generated. Figure 3 describes the capture of high refractive index nanoparticles by different numbers of arrays of Gaussian beams. The figure illustrates the gradient force acting on the nanoparticles along the x-direction in the transverse plane, which is located 0.5 μm away from the focal plane along the z axis, under different values of n and different translation distances. The number of optical traps generated in the transverse plane is only related to the input light field and is independent of the gradient force of the optical traps. For multiple optical wells formed by the array of Gaussian beams, if *n* > 2, the generated optical arrangement is a regular n-polygon. In the optical trap formed by two beams in Figure 3B, the gradient force points at the focus of one beam, and the cross force is generated by the scattering light field caused by the other beam. If any beam in the double optical trap deviates from its equilibrium position, the amplitude of the scattering light field generated by the other beam is modulated. Therefore, the cross force tends to correlate with the motion of the trap. Moreover, an array of Gaussian beams is expected to be applied to the random motion cooling of the center of mass of the sphere.

**Figure 4:**
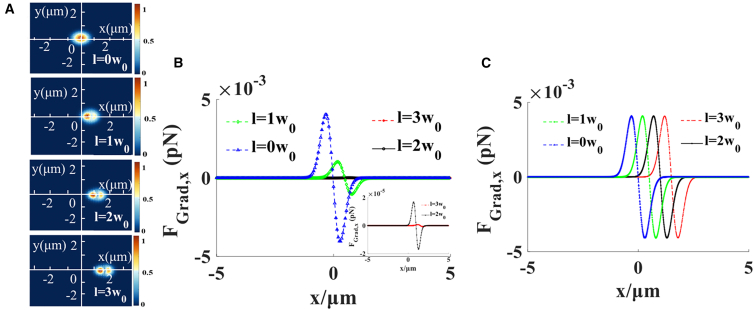
Gradient force of an array of Gaussian beams with *n* = 1 for different translation distances *l* at Δz = 0.5 μm, Δz is the distance from the focal plane along the z axis (A) Illustrates different translation distances *l*. Moreover, when taken as a positive integer, the beam generated on the focal plane gradually moves to the right. (B) Indicates that as the translation distance *l* increases, the gradient force at the focal point gradually decreases. (C) Indicates that the translation of the beam does not affect the magnitude of the gradient force at the center of the beam.

Figure 4 illustrates a gradient diagram of particle capture by an array of Gaussian beams at Δz = 0.5 μm with different translation distances *l* when *n* = 1, The parameter Δz denotes the axial distance between the output plane and the focal plane along the z axis. As shown in Figure 4B, with an increase in the distance *l*, the faculae of Gaussian beams on the output plane move further from the focus, and less light is generated at the focus. However, the light force of each Gaussian beam is the same at the center, which is evident in Figures 4A and 4C. Figure 4C indicates that the value of each optical gradient force in the transverse direction is the same at the focus, and the position of the capture point changes with the shift distance. In any plane along the beam axis, particle capture can be controlled by controlling the capture position in the optical trap.

**Figure 5:**
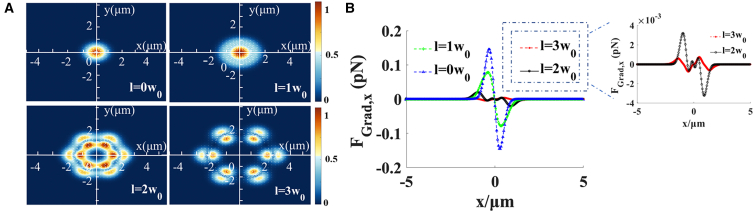
Evolution of the gradient force field distribution of an array of Gaussian beams (*n* = 6) passing through a focusing system at a distance from the focal plane (A) Shows that as the translation distance *l* increases, the array of Gaussian beams outside the focal plane gradually disperses and is arranged in a regular hexagonal shape. The gradient plot in (B) shows that the gradient force decreases with increasing translation distance. (A dynamic diagram is attached in the supplementary materials (Video S1 (Related to (A)).

Figure 5 shows a trend diagram of the array of Gaussian optical traps for six beams gradually generated at Δz = 0.5 μm by changing the translation distance *l*. With the increase in *l*, the value of the optical gradient force at the focus decreases, and the six array of Gaussian beams become easy to distinguish, as shown in Figure 5A. (A dynamic diagram is attached in the supplementary materials (Video S1 (Related to Figure 5A). This result also verifies the light intensity evolution analysis in Figure 3. Figure 5B clearly shows that the optical trap stiffness of an array of Gaussian beams with *n* = 6 differs at different translation distances. Trap stiffness is a physical quantity that characterizes the mechanical properties of optical tweezers, and it also acts as a metric for describing the response of the trap to the displacement of trapped nanoparticles away from the trap center. The magnitude of the force exerted by the optical field on the trapped nanoparticles exhibits a linear correlation with the particles’ displacement relative to the trap center; the proportionality constant in this linear relationship is thus defined as the trap stiffness. By adjusting the translation distance, we can adjust the trap stiffness of an array of Gaussian beams, which has research potential in ultra-high precision optical force and acceleration sensing.^39^

Video S1. The gradient force field of the array of Gaussian beams (n = 6) varies with the translation distance

**Figure 6:**
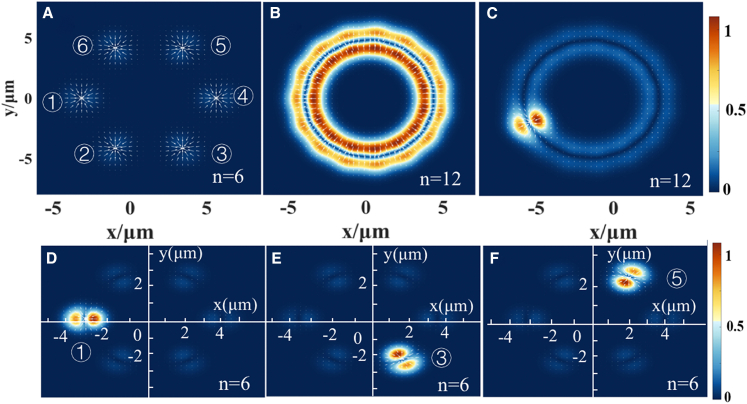
Flexible control of an array of Gaussian beams (*n* = 6) and a modulating array of Gaussian beams to generate a hollow beam (A) Shows the gradient force field of an array of Gaussian beams (*n* = 6) when G_6_ is an all-one matrix. (D–F) Show the gradient force field of an array of Gaussian beams (*n* = 6) when the G values of single beams 1, 3, and 5 are greater than those of the other 5 beams. (B) When n is large, the array of Gaussian beams (*n* = 12) generates a positive dodecagon configuration, generating a hollow beam gradient field. (C) Shows that a cyclic assignment of the G-value matrix for the array of Gaussian beams (*n* = 12), can cause the captured beam to rotate. (A dynamic diagram is attached in the supplementary materials (Video S2 (Related to (C)).

Figures 6A–6D and 6F show that the array of Gaussian beams for *n* = 6 can be adjusted independently. We can enlarge the gradient force of any one of the beams to control the nanoparticles flexibly. For example, if we select the array of Gaussian beams (*n* = 6) and pick the 1st, 3rd, and 5th beams in sequence, the intensity of each selected single beam is higher than that of the other beams, with the amplified single beam carrying three times the intensity of the latter. According to the formula in Equation 9, the following Figures 6D–6F can be obtained.

(Equation 9) $$G_{6}=\left[\begin{array}{cccccc} 3 & 1 & 1 & 1 & 1 & 1\\ 1 & 1 & 1 & 1 & 1 & 1\\ 1 & 1 & 3 & 1 & 1 & 1\\ 1 & 1 & 1 & 1 & 1 & 1\\ 1 & 1 & 1 & 1 & 3 & 1\\ 1 & 1 & 1 & 1 & 1 & 1 \end{array}\right]\;G_{12}={\left|\begin{array}{ccccc} 3 & 1 & 1 & \cdots & 1\\ 1 & 3 & 1 & \cdots & 1\\ 1 & 1 & 3 & \cdots & 1\\ \vdots & \vdots & \vdots & \ddots & \vdots \\ 1 & 1 & 1 & \cdots & 3 \end{array}\right|}_{12\times 12}$$

Array of Gaussian beams can be modified into new beam shapes by adjusting the number of optical wells, such as by forming hollow optical wells, as shown in Figures 6B and 6C. By controlling the number of optical wells n and translation distance *l*, a regular polygonal array of Gaussian beams can be generated. For example, consider *n* = 12 and *l* = 3*w*_0_, as shown in Figure 6B. This beam is very dense, and a hollow beam with zero middle light intensity can be generated. At this time, if the nanoparticles are captured on the ring, we can use the *G*_12_ matrix in Equation 9 by cyclically modulating the intensity of every single beam of a Gaussian array beam, and the nanoparticles can be rotated by continuously changing a single beam, as shown in Figure 6C. (A dynamic diagram is attached in the supplementary materials (Video S2 (Related to Figure 6C). The speed of particle rotation is dependent on the frequency of the phase modulator. For example, if a spatial light modulator is used, the rotation rate can reach the order of kilohertz. We provide a new method of rotating and manipulating nanoparticles.

Video S2. Trapping and rotation of nanoparticles using array of Gaussian beams

**Figure 7:**
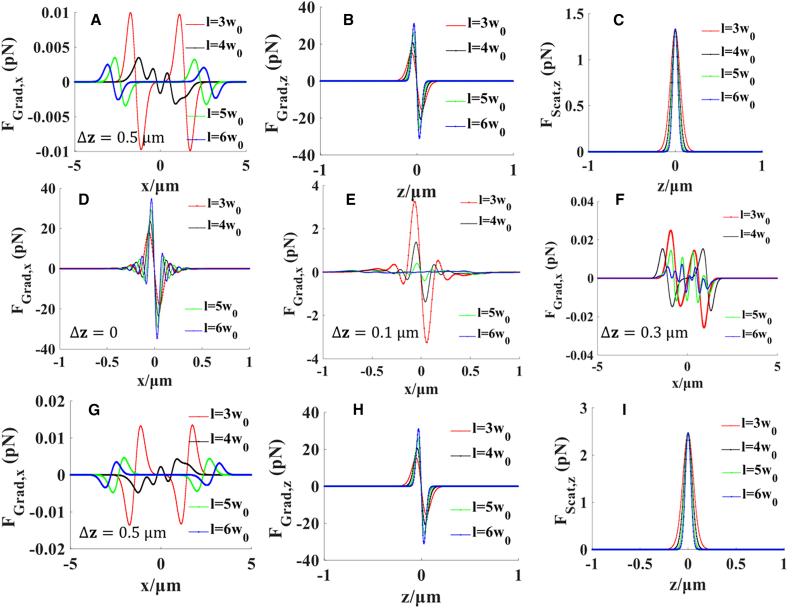
The distribution of the radiation force on the nanoparticles Radiation force of two kinds of nanoparticles with (A–F) high and (G–I) low refractive indices at different translation distances *l* by an array of Gaussian beams We selected a nanoparticle with a radius *r* = 20 nm; *n*_*m*_ = 1.332 is the refractive index of the surrounding field, and the high and low refractive indices are the homogeneous Rayleigh nanoparticles. Other parameters are *λ* = 1.064 μm, *w*_0_ = 5 mm*, f* = 5 mm, P = 1 W, and G_12_ is set as an all-one matrix.

Figures 7A–7C show a radiation force diagram of nanoparticles in an optical trap formed by positive dodecagon beams when the refractive index of the nanoparticles is 1.592 (i.e., polystyrene), and the translation distance is 3*w*_0_, 4*w*_0_, 5*w*_0_, and 6*w*_0_. Figure 7A illustrates the distribution of the transverse gradient force along the *x*-direction on the output plane at Δz = 0.5 μm from the focal plane. The hollow area of the hollow beam increases, and the overall gradient force decreases with the increase in the translation distance. In Figure 7A, when the translation distance is 4*w*_0_, the transverse gradient force fluctuates, indicating that the regular dodecagon beams disturb each other. At this time, owing to the existence of coupling forces, the stiffness of the optical trap changes rapidly, and a hollow optical trap cannot be formed. Figures 7B and 7C show the gradient and scattering forces on the axis at different translation distances. From the gradient and scattering diagrams, with the increase in the translation distance *l*, the capture area of the radial gradient force evidently reduces, and the scattering area of the scattered light decreases. As shown in these diagrams, the axial gradient force is greater than the scattering force. According to the fluctuation-dissipation theorem of Einstein, the magnitude of the Brownian force is expressed as $F_{b}=\sqrt{12\eta ak_{B}T}$ ,where *η* = 7.977 × 10^−4^ *Pa*. *s* is the viscosity for water at the room temperature T = 300 K, the Brownian force is *F*_*b*_ ≈ 1.58 × 10^−15^*N*, the gradient force acting on the nanoparticle in the optical trap is greater than the Brownian force, thus, nanoparticles with a high refractive index can be stably captured at the bright ring of the hollow beam. Figures 7D–7F illustrate the numerical variation in the transverse gradient force along the *x*-direction at defocus distances ranging from 0.1 μm to 0.5 μm from the focal plane, where the gradient force decreases rapidly with increasing distance. As can be seen from Figures 7D and 7E, the array of Gaussian beams exhibits Gaussian beam characteristics at the focal plane, enabling the trapping of high-refractive-index nanoparticles at the focal point. Moreover, it can be inferred from Figures 7A and 7D–7F that the farther the distance from the focal plane, the smaller the transverse gradient force.

Figures 7G–7I present the radiation force formed by the same beam when the refractive index of the nanoparticles is low. Figure 7G indicates that low-refractive-index nanoparticles (i.e., water) can be trapped by the transverse gradient force on the plane 0.5 μm away from the focus. Similarly, when the translation distance is 4*w*_0_, the lateral gradient force fluctuates, reducing the capture area. However, the gradient force capture capability is not affected. In the radial direction, with an increase in the translation distance *l*, the gradient force becomes smaller while the capture area becomes larger. Figures 7H and 7I clearly show that the gradient force of low-refractive-index nanoparticles in the radial direction is greater than the scattering force. Thus, this hollow beam can stably capture low-refractive-index nanoparticles.

## Discussion

We introduced a model to generate a flexible and controllable array of Gaussian beams and studied the focusing characteristics of the beam. Upon modulating the parameter n and translation distance *l*, our results clearly show the different characteristics of the array of Gaussian beams and the previous multi-beams. We demonstrate the flexible joint control and modulation of multiple beams within an optical trap via a single specialized beam, and extend its application to the rotational manipulation of nanoparticles. An Array of Gaussian beams can adjust the capture area of nanoparticles, generate distinct beam profiles, and capture two kinds of particles with different refractive indices in the optical trap simultaneously. The capability of this beam to achieve axial manipulation and rotation of micro-nanoparticles can fulfill sample scanning requirements, thereby eliminating the dependence on mechanical translation stages in optical tweezers setups. Leveraging the non-contact and non-destructive manipulation mode of optical tweezers, this work can promote interdisciplinary research integrating optical tweezers with other microscopy techniques, showing potential for applications in cell biology and medical fields.

### Limitations of the study

The article constructs the optical field of an array of Gaussian beams with arbitrary regular polygonal arrangements by employing the action of cyclic groups and rotational transformations on Gaussian functions. As presented above, varying the approaches for the central translation and rotational transformation of Gaussian beams allows the generation of an array of Gaussian beams that can be extended to arbitrary geometric shapes. Further research can be carried out on such an array of Gaussian beams with arbitrary geometric shapes, which can further facilitate the integration and practical application of metasurface-based optical tweezers systems.

## Resource availability

### Lead contact

Requests for further information and resources should be directed to and will be fulfilled by the lead contact, Hui-Zhu Hu (huhuizhu2000@zju.edu.cn).

### Materials availability

This study did not generate new unique reagents.

### Data and code availability

• Data reported in this article will be shared by the lead contact upon request.
• This article does not report original code.
• Any additional information required to reanalyze the data reported in this article is available from the lead contact upon request.

## Acknowledgments

This work was supported by the Fundamental Research Funds for the Central Universities [grant number 2018XZZX001-08], and 10.13039/501100001809National Natural Science Foundation of China [grant number 62075193].

## Author contributions

J.S. wrote the main article text. N.L. and C.J. performed the statistical analysis. X.C. and H.H. prepared figures. All authors contributed to article revision, read, and approved the submitted version.

## Declaration of interests

The authors declare no competing interests.

## STAR★Methods

### Key resources table

**Table:** 

REAGENT or RESOURCE	SOURCE	IDENTIFIER
**Software and algorithms**		

MATLAB	MathWorks	https://www.mathworks.com/products/matlab.html

### Experimental model and study participant details

This study does not use experimental model and subject details typical in the life sciences.

### Method details

Based on the Collins formula derived from paraxial scalar diffraction theory, the paper derives the propagation and trapping expressions of the array of Gaussian beams in a focusing system and simulates the optical and gradient field distributions for different parameters. By tuning the translation factor, number of beams, and other parameters, the array of Gaussian beams enables beam shaping and rotational particle trapping. All radiation force models are implemented using MATLAB R2025a.

### Quantification and statistical analysis

This study does not include quantification and statistical analysis.
